# The Predator–Prey Encounter Detection (PrED) Model: Detecting Interactions Between Rotifers and Algae Using Computer Vision and Machine Learning

**DOI:** 10.1002/ece3.74286

**Published:** 2026-09-06

**Authors:** Braden Charles DeMattei, Stephanie E. Hampton

**Affiliations:** ^1^ Department of Evolution, Ecology, and Organismal Biology The Ohio State University Columbus Ohio USA; ^2^ Tahoe Environmental Research Center University of California Incline Village Nevada USA

**Keywords:** algae, computer vision, machine learning, plankton, predator, prey, rotifer, species interactions

## Abstract

Understanding species interactions is integral to understanding ecosystem function and predicting how disturbances may affect the services provided to society. Analysis of trophic interactions, however, requires a significant investment of time and resources. Using a simple predator–prey pairing of a semi‐sessile rotifer and motile algae, the Predator–Prey Encounter Detection (PrED) model aims to streamline and semi‐automate video analysis of species interactions using computer vision and machine learning techniques. Based on Ultralytics YOLOv5 architecture with an implementation of SORT tracking, the PrED model can estimate algal density and generate a “potential encounter” log with timestamps to allow researchers to skip through a video. After the model validation and testing steps, the PrED model achieved over 90% precision and recall scores. By reducing the time it takes to analyze a species interaction video, the PrED model will save researchers time and act as an efficient tool to study plankton dynamics.

## Graphical abstract

Using a simpler predator‐prey pairing of a semi‐sessile rotifer and motile algae, the Predator‐Prey Encounter Detection (PrED) model aims to streamline and semi‐automate video analysis of species interactions using computer‐vision and machine learning techniques. Based on Ultralytics YOLOv5 architecture with an implementation of SORT tracking, the PrED model can estimate algal density and generate a “potential encounter” log with timestamps to allow researchers to skip through a video. By reducing the time it takes to analyze a species interaction video, the PrED model will save researchers time and act as an efficient tool to study plankton dynamics.

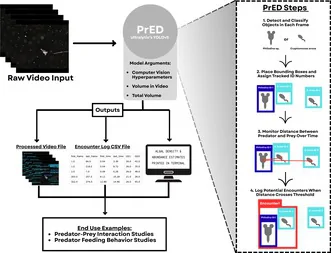

## 1 Introduction

Species interactions are integral to the functioning of ecosystems and the services they provide society (Traill et al. 2010). As environmental change alters these systems, interactions between species also change, affecting the way ecosystems function overall (Traill et al. 2010; Wollrab et al. 2021). Scientists and natural resource managers require robust information about species interactions in order to understand and predict ecosystem change, yet a comprehensive, in‐depth understanding of community dynamics requires substantial resources and time (Pringle and Hutchinson 2020). Mechanistic studies of species interactions, such as those of predators and prey, typically require high‐resolution data produced from detailed field and lab experiments or molecular approaches that are expensive, system specific, or both (Pringle and Hutchinson 2020). Quantifying species interactions from video datasets typically relies on labor‐intensive methods such as scanning hours of video to conduct behavior scoring (encounter, attack, retention, rejection, ingestion) on each individual interaction (Salt 1987). In addition to the complexities these methodological hurdles present, a given ecosystem's community dynamics can vary across several spatial and temporal scales, making it even more onerous to fully capture predator–prey interactions (Carroll et al. 2019; Pringle and Hutchinson 2020; Bandara et al. 2021; Suraci et al. 2022; Jiang et al. 2023). In this time of rapid environmental change, it is important to streamline the methods and analyses used in community ecology to make it easier to understand an ecosystem's baseline community dynamic and use it as a reference point to record how these systems are adapting to new environmental trends.

One way to ease the burden of current species interaction analysis methods is by incorporating new tools such as automated image analysis, or Computer Vision, into ecological studies. To date, computer vision and machine learning (CV/ML) applications in ecology have mostly focused on the detection, classification, and tracking of species (Weinstein 2017; Waldchen and Mader 2018). These studies demonstrate the great potential for automated and semi‐automated tools to be specifically developed for and applied to major questions in species interactions, community ecology, and food web dynamics in a greater variety of systems (Tresson et al. 2019). The full realization of automated detections of predator–prey interactions would radically advance food web research and allow for a deeper, more comprehensive understanding of the community dynamics that underlie ecosystem functioning across systems.

Microscopic aquatic predator–prey systems provide an ideal testbed in which to develop and advance CV/ML workflows. Predator–prey interactions occur within a few mL of water over scales of seconds to minutes, observable by microscopy, and with broad potential for investigators to manipulate conditions. Here we report on a rotifer‐algae system; this “simple” predator–prey system opens up the possibility of not only improving food web understanding in aquatic systems but also improving computer vision applications more broadly. Using rotifers and algae as the initial species creates a base for understanding how CV/ML models detect nuanced interactions between taxa, as their behaviors are simplistic compared to many macroscopic organisms. An initial key step in the development of generalizable computer vision models that detect and classify predator–prey interactions is to develop a CV/ML model that detects encounters.

This study focused on the development of a model that can differentiate between specific predators (rotifers) and prey (algae), and detect when the two encounter each other. An encounter here is defined as the distance at which the rotifer's chemosensory receptors detect an algal cell, which to the human eye appears as if the alga and the rotifer's corona are coming into physical contact (Gilbert and Stemberger 1985; Salt 1987). With this definition in mind, this model logs every potential encounter and its related timestamp into an output file so that the user can quickly jump to those moments and efficiently determine the number of encounters that occurred in the video. As the number of encounters in a given time period is influenced by prey density (algal cells per mL) and abundance (total algal cells), the model also has the ability to roughly estimate these contextual metrics (Ioannou et al. 2008). This model should not be considered fully automated as it does not estimate encounter frequency nor the more complex effects of microturbulence and prey relative velocity on this rate (Gerritsen and Strickler 1977; Rothschild and Osborn 1988). It, therefore, does not replace the need for human analysis and calculation. What this model does do, however, is give the user a list of time frames that may be of interest, avoiding the need to scrub through a video frame by frame. Simply put, the Predator–Prey Encounter Detection model (PrED) serves as a helpful, time‐saving video analysis tool that can estimate algal density and abundance and generate a log of potential predator–prey encounters for later review and use in encounter rate analysis.

This study should also serve as a road map for other ecologists wishing to learn about and incorporate CV/ML methods into their work who otherwise may have limited computer science experience. The model presented here was developed by ecologists with no prior CV/ML experience, so the authors aim for this study to give readers the knowledge and confidence to integrate these cutting edge tools and techniques into their research. The scripts used for this model are written in the Python programming language allowing it to be operating system agnostic as well as highly adaptable for users' needs (version 3.12; Python Software Foundation, n.d.). While this model focuses on rotifers and algae, others wishing to use the general PrED model pipeline to expedite video analysis of their chosen species pairings simply need to train the model on their own annotated video frames and adapt the inference scripts to their needs. These steps are described below. As CV/ML tools advance and more become available, we hope this study demonstrates that ecologists can and should add these emerging methods to their tool box with confidence.

## 2 Methods

### 2.1 Predator–Prey System

The bdelloid rotifer *Philodina* sp. Ehrenberg, 1830 was used as the predator in the development of PrED. They are semi‐sessile, greatly facilitating observation of the predator for the initial development of our model. These rotifers propel themselves through the water using ciliary motion of their corona, and they frequently anchor themselves to the substrate using their foot, presumably in response to a food patch or another favorable circumstance. Once anchored, an individual uses its corona to create a current in the water that brings its prey to it (Starkweather 1995). The prey used was the algal species *Cryptomonas erosa* Ehrenberg, 1832, a motile alga that propels itself through the water using two flagella. As the algae swim through the water, they become entrained in the rotifer's feeding field and encounter the rotifer's perceptual field in the area of the corona. This predator–prey pairing facilitated this initial stage of model development by eliminating some of the common difficulties associated with filming aquatic species. For example, the predator remaining stationary allowed for large portions of its hunting effort to be recorded without needing to refocus the microscope or move the well‐plate. This stationary setup resulted in a fixed depth of focus and magnification which also allowed for estimation of prey density and abundance.

### 2.2 Culturing

We collected bdelloids from the lily pond next to the Mabel and Arnold Beckman Laboratories of Behavioral Biology at California Institute of Technology (Pasadena, CA, USA) in June 2024. Water for culturing was collected from the pond and vacuum‐filtered through a GF/F filter. We started the culture with a single bdelloid rotifer transferred to a petri dish and allowed to reproduce *ad libidum* with *C. erosa* . Cultures were split every three days to keep densities uncrowded in cultures, with fresh culture medium and food. The *C. erosa* culture was a strain well known to promote rotifer growth, in continuous culture for over 40 years (Stemberger 1981), and obtained from Dr. Elizabeth Walsh's lab at University of Texas, El Paso, cultured in UTEX WC medium.

### 2.3 Video Collection

The videos were captured at 25 frames per second using Zeiss ZEN lite and a Zeiss SteREO Discovery v20 dissection microscope with an Axiocam 705 Color under dark‐field conditions. A PlanApo S 0.63× objective was used with the zoom set to 120×, which created a field of view with an area of 0.0241 mm^2^. Using a 12 well culture plate, 0.8 mL of GFF filtered pond water was added to a single well via a serological pipette. A single bdelloid rotifer was isolated and transferred to the well using a fine tipped glass pipette to minimize introduction of additional liquid. Once the bdelloid attached itself, 0.2 mL of the *C. erosa* culture was added and recording began. The recording continued until either the rotifer retracted its corona for more than 5 s or the software slowed down substantially. The latter was the most common reason to stop recording, signaling an impending software crash.

### 2.4 Dataset & Model Preparation

Altogether, 25 videos were taken with a total of 45,158 frames, with the frame count of a given video ranging from 550 to 4000 frames. The videos were exported onto an external hard drive and compressed AVI files were later converted to MP4 files using “ffmpeg” so that they would be playable on a Mac operating system. The files were uploaded to Label Studio (version 1.18; Tkachenko et al. 2020–2025) for annotation. Frame by frame, investigators labeled the rotifer (class: “bdelloid”) and each individual *C. erosa* (class: “cerosa”) using bounding boxes. Since the *Cryptomonas* could swim up and out of the focal plane, we determined that they were occluded if they began to “donut” (i.e., become so out of focus they look like a red ring with a dark spot in middle). After the initial round of annotation, investigators watched the video at normal speed to check for mistakes or unlabeled algae. Once all the videos were labeled, the annotations were exported in the Label Studio format in a JSON file. Using a Python (version 3.12; Python Software Foundation, n.d.) script, each frame's set of labels were taken out of the JSON file, converted to the YOLO coordinate format, and stored in a text file named after the frame (Video1_001.txt, e.g.). The videos were then divided into training (33,559 frames, 18 videos), validation (7139 frames, 5 videos), and test (4420 frames, 2 videos) sets. This roughly followed the 80%–10%–10% split Ultralytics recommends when preparing a dataset for use with YOLOv5 (Ultralytics 2025).

Using Ultralytic's YOLOv5 code architecture, the model was trained using the YOLOv5 nano pre‐trained weights (Ultralytics 2025). The image size for each frame was set to 1920, so that the resolution was high enough for the *C. erosa* to be retained in each frame, but low enough to keep training time to a minimum. Albumentations (version 2.0.8; Buslaev et al. 2018) was implemented into the training script to add random augmentations to the dataset before training commenced to generalize the training set further by creating “new” images on the fly from the existing training set (Table 1). The number of training epochs was set to 300, with patience set to 20 epochs. An epoch here means one round of the model going through every image in the training dataset, while patience is a parameter that looks at whether the model continues to improve across epochs and terminates training if it is not. In this case, training terminated at 24 epochs, meaning that the loss calculated during the validation step was not improving past epoch 3. Loss, in a computer vision context, represents how wrong the model is compared to the ground truth of the annotated dataset; loss is what the model is trying to minimize across epochs. The default loss functions were used for the three main tasks the model was performing: bounding box placement, object detection, and object classification (Table 2). Several combinations of model size (nano vs. small pretrained weights) as well as patience and epoch settings were used for training, but the hyperparameters reported here resulted in the lowest loss and highest Precision, Recall, and Mean Average Precision (mAP) values.

**TABLE 1:** List of the image transformations and their associated parameters used when training the model.

Transformation	Parameters
Blur	*p* = 0.01, blur_limit = (3, 7)
MedianBlur	*p* = 0.01, blur_limit = (3, 7)
ToGray	*p* = 0.01, method = “weighted_average”, num_output_channels = 3
CLAHE	*p* = 0.01, clip_limit = (1.0, 4.0), tile_grid_size = (8, 8)
RandomBrightnessContrast	*p* = 0.1, brightness_by_max = True, brightness_limit = (−0.2, 0.2), contrast_limit = (−0.2, 0.2), ensure_safe_range = False
RandomGamma	*p* = 0
ImageCompression	*p* = 0.1, compression_type = “jpeg”, quality_range = (99, 100), quality_lower = 75
RandomResizedCrop	*p* = 0

*Note:* These transformations come from the Albumentations package. RandomGamma and RandomResizedCrop were not used and were therefore set to zero.

**TABLE 2:** Loss functions used when evaluating the PrED model on the three computer vision tasks it performs.

Task	Loss function
Box placement	Generalized intersection‐over‐union
Classification	Cross‐entropy
Object detection	Binary cross‐entropy

*Note:* The loss functions listed here are the defaults set for YOLOv5 by Ultralytics.

### 2.5 Modifications to YOLOv5 Inference Script

**FIGURE 1:**
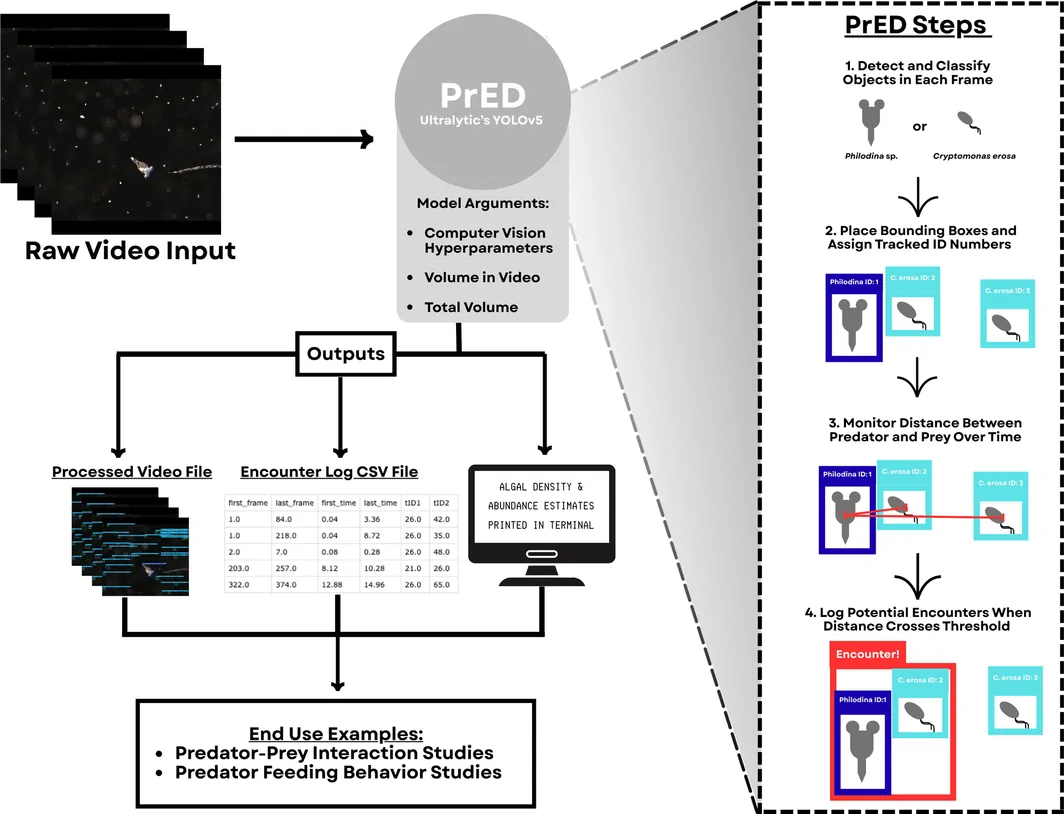
A conceptual diagram showing the Predator–Prey Encounter Detection (PrED) model workflow starting with the raw video input, then the model's outputs, and ending with potential end use cases. The inset shows the PrED model's internal pipeline when processing the raw video input.

While Ultralytic's YOLOv5 default training script was used for the steps described above, it was necessary to modify the default inference python script (“detect.py”) to add the following functionalities: tracking, encounter detection, and prey density estimation (Figure 1). For tracking, the Simple Online and Realtime Tracking (SORT; Bewley et al. 2016) model was implemented into the modified inference script (“pred_detect_sort.py”). SORT assigns each object with a bounding box a unique identifier and then predicts its trajectory across frames. SORT was instrumental for the encounter detection functionality in PrED. Taking SORT's unique identifiers, the inference script differentiates each ID by class and measures the normalized distance (nDist) between every *C. erosa* to the closest *Philodina* sp. bounding box. For rotifers, and specifically for *Philodina* sp., encounters are noted when the prey item comes close to the corona after getting caught in the rotifer's feeding field, which to the human eye looks like physical contact (Gilbert and Stemberger 1985; Salt 1987). Thus the nDist threshold for this predator–prey pairing was set to < 0.1, which corresponded to the distance where an algal cell becomes entrapped in the field and was brought into contact with the corona. Because the distance for which an encounter is recorded can differ among predatory species, the normalized distance threshold for an encounter was included as an argument (“threshold”) in the modified inference script, allowing users to set an appropriate value for their own species pairing.

**FIGURE 2:**
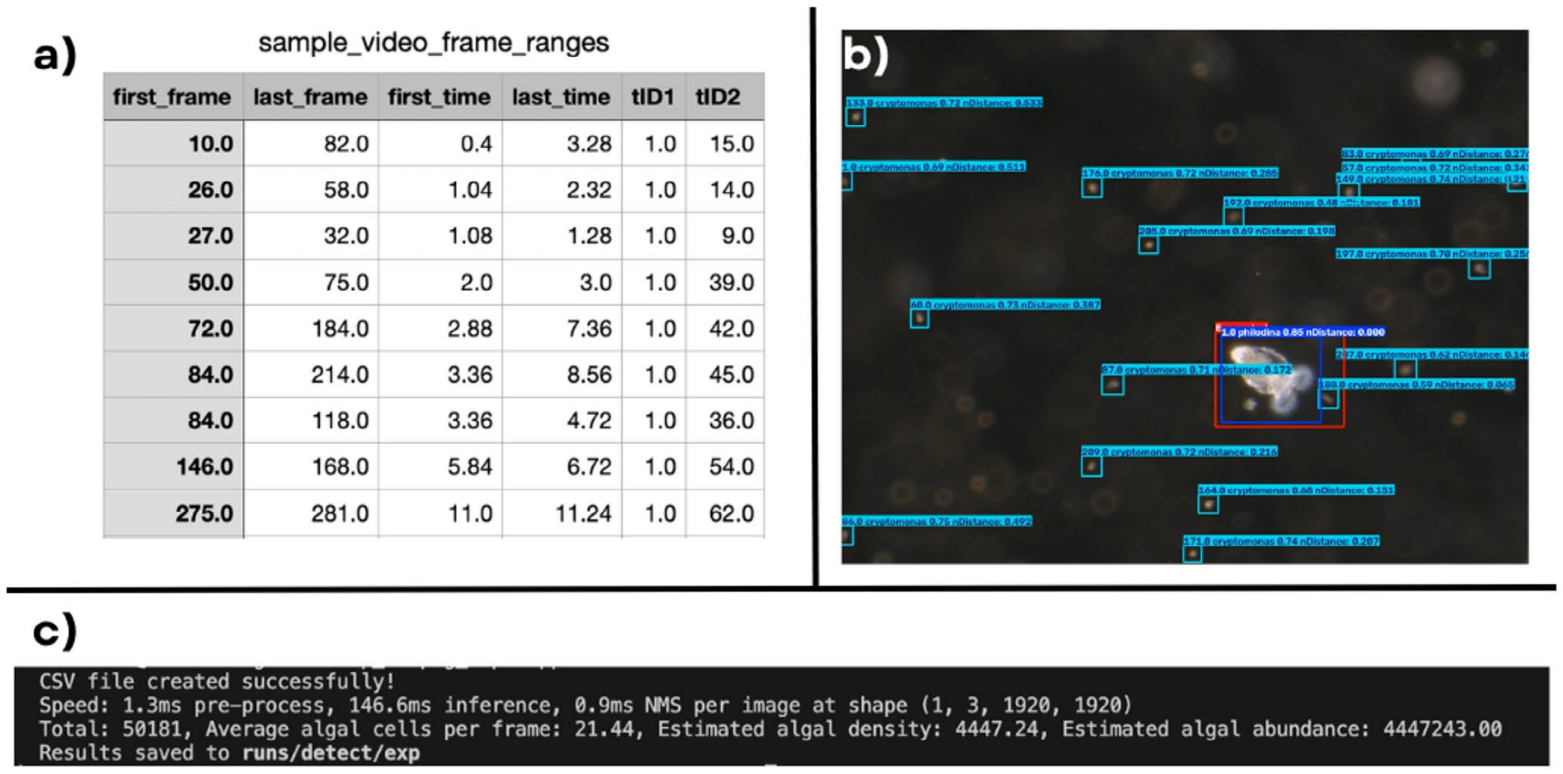
Example outputs from “pred_detect_sort.py” run on a sample video with 2341 frames. (a) The potential encounter log showing the first frame, last frame, first time, last time, and the tracked ID numbers for the two individuals involved in the encounter. (b) An example frame from the processed video with bounding boxes, including the red “Encounter!” box indicating a detection of a potential encounter. (c) The terminal output printed after the script finished running which includes the total number of algal cells seen across all frames, the average number of cells per frame, as well as the estimated density and abundance values. This also includes the confirmation messages that files were saved as well as the time each step of the process takes per image: pre‐process, inference, and post‐processing (NMS). Adding these three time values together and multiplying by the number of images will yield the amount of time in milliseconds it took to process one video.

Once the set threshold is reached, the script adds a new, red “Encounter!” bounding box around the two objects (Figure 2b). This encounter is then logged into an output file where the start frame, end frame, start time, end time, and the two tracked IDs involved in the encounter are stored. This output file is saved into the parent directory and named “[VIDEO TITLE]_frame_ranges.csv” (Figure 2a). The script also tracks the number of *C. erosa* the model sees for each frame and keeps a running total. Once every frame has been processed, this total is divided by the total number of frames in the video to find the average number of individuals seen per frame. This value is divided by the volume of the viewing plane (*V*_*a*_ = 0.00482 mm^3^) to find a rough estimate of the prey density, assuming uniform density. The volume of the viewing plane, *V*_*a*_, was calculated by multiplying the area of the viewing plane, 0.0241 mm^2^, by the length of the average *Philodina* sp., 0.2 mm. The estimated total abundance in the well (*V*_*T*_ = 1000 mm^3^) was found by multiplying the prey density estimate by *V*_*T*_. These values are then printed in the terminal when the inference script has finished running (Figure 2c). When executing the inference script, the best model weights (“weights/best.pt”) from training were used and the confidence value was set to 0.1, meaning that the model's confidence that an object was of a certain class needed to be ≥ 0.1.

## 3 Model Evaluation

**FIGURE 3:**
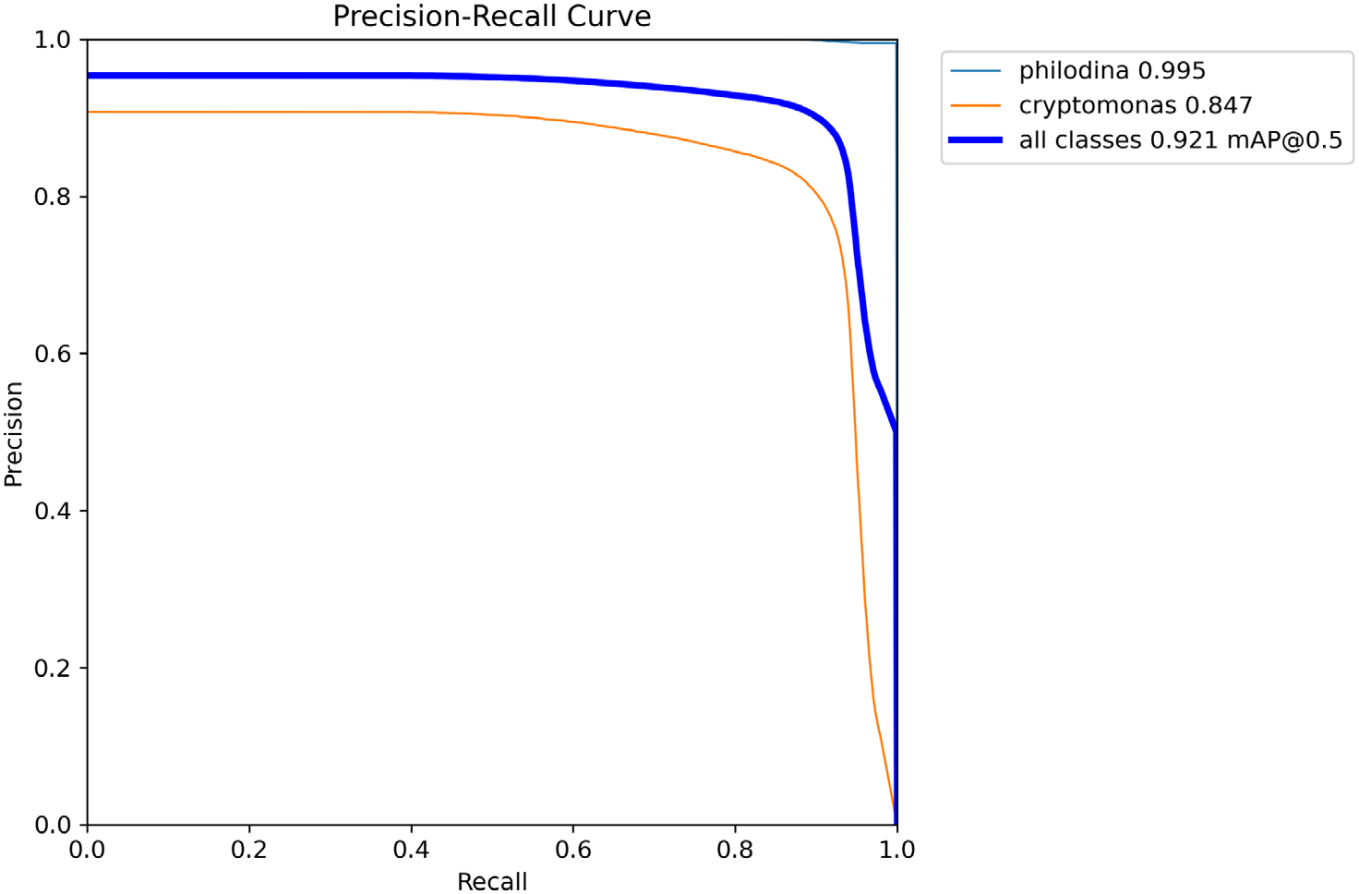
The Precision‐Recall (PR) Curve automatically generated by YOLOv5. These curves represent precision and recall values under different confidence thresholds for detections to be triggered. Precision and recall are inversely related as confidence thresholds increase. For Philodina, the curve is difficult to see as its precision and recall are both roughly equal to 1 across all confidence thresholds, with the average precision being 0.995. Thiscurve can be seen in the upper right corner of the plot. For Cryptomonas, precision stays steady across all confidence thresholds, until recall reaches 0.8, where it begins to drop. The model's average precision specifically for Cryptomonas across confidence thresholds is 0.847. The blue line represents the mean Average Precision at a 50% IOU threshold (mAP50) and is 0.921 across both classes.

The model was evaluated based on Precision (P), Recall (R), mean Average Precision with an Intersection over Union (IOU) threshold of 0.5 (mAP50), and mAP with various IOU thresholds between 0.5 and 0.95 (mAP50:95). Precision represents how accurate the model is when it makes detections, and is the proportion of correct detections to all detections. Recall, on the other hand, represents whether the model found all the possible instances of objects annotated in the dataset, and is the proportion of correct detections to the total number of true detections that the model could have made. Mean Average Precision is found by averaging the areas under the Precision‐Recall curve over varying Recall values, with IOU representing the amount of overlap between the bounding box placed by the model and the bounding box placed during annotation (Lin et al. 2015; Figure 3). The mAP values are meant to act as an overall evaluation score of the model for a given dataset, and the higher they are the better the model is at detecting objects (Lin et al. 2015). For mAP50, the IOU is fixed at 0.5 (meaning the bounding boxes share 50% of the same area) and detections are only considered correct when the IOU is equal to or greater than 0.5 (Lin et al. 2015). The mAP50:95 metric evaluates the model more stringently by calculating mAP values at multiple IOU thresholds between 50% and 95% overlap and then averages them together (Lin et al. 2015).

**FIGURE 4:**
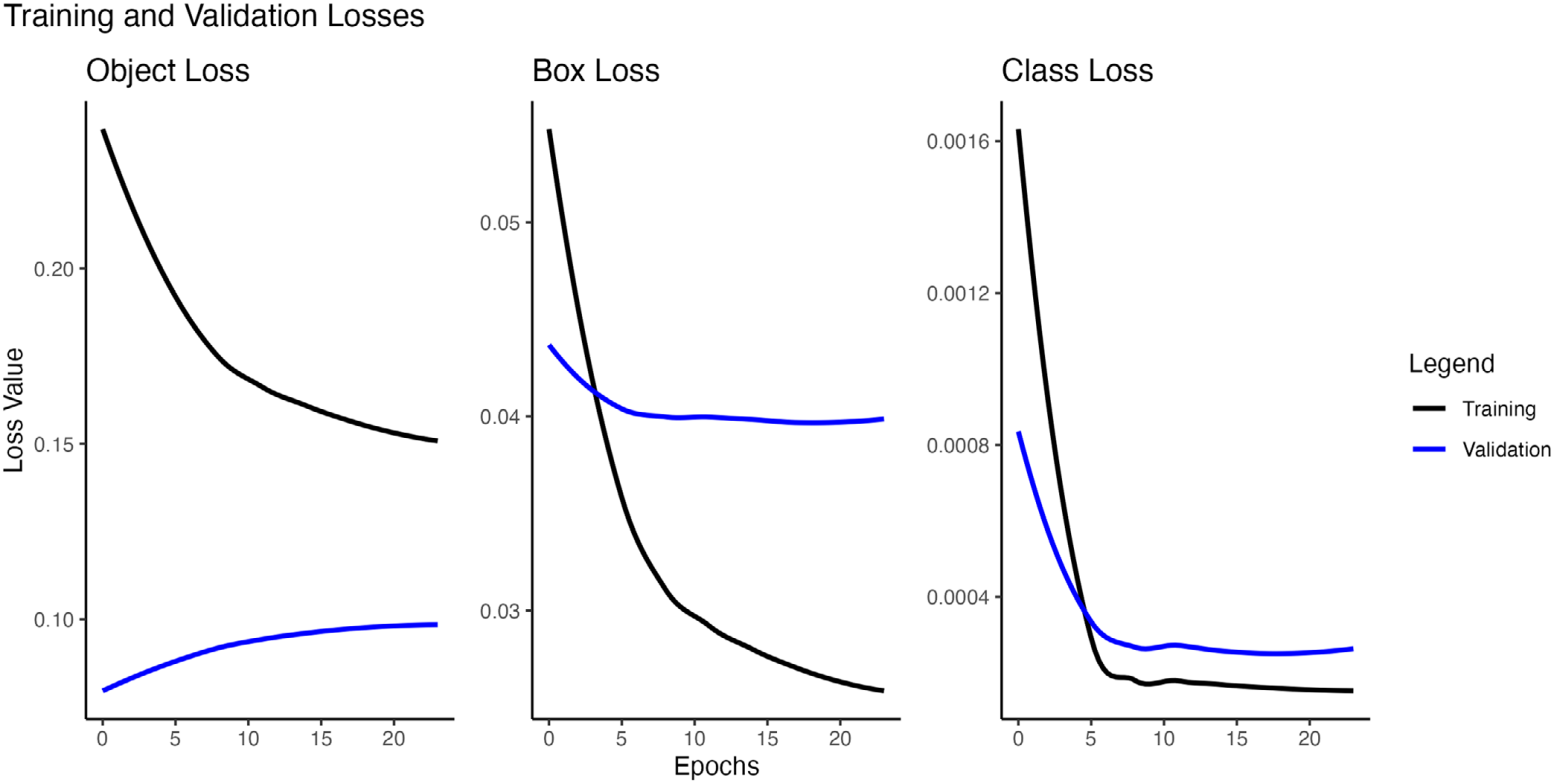
The training and validation loss smoothed curves for object detection, bounding box placement, and object classification. Note the differences between the y‐axis scales among the three graphs. The model improved in all three tasks during training (black lines) as can be seen in the decrease in loss across all three graphs. For validation (black lines) however, the model showed slight improvement for the bounding box and classification tasks, while it showed a slight increase in loss for object detection.

**FIGURE 5:**
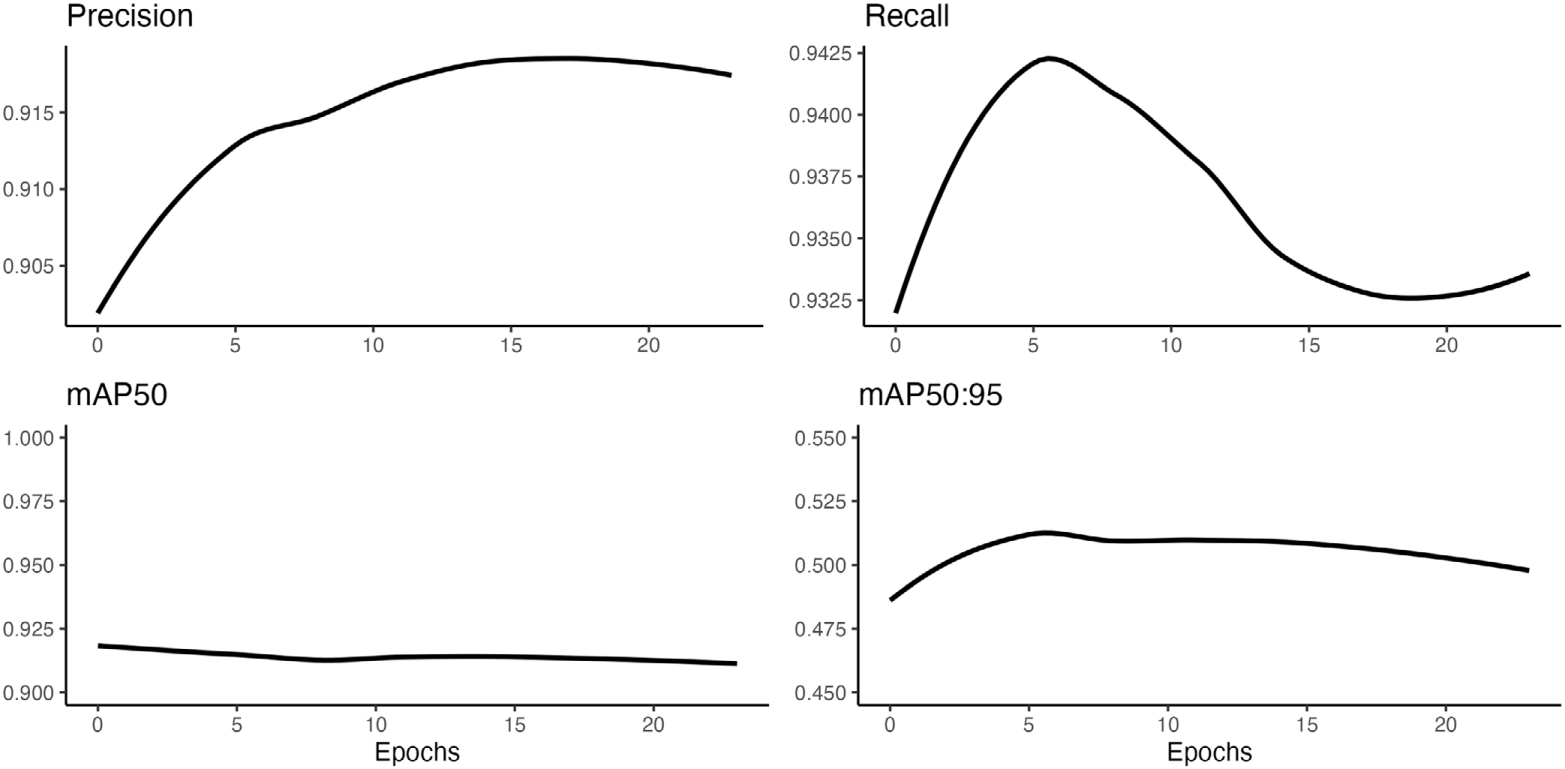
The Precision, Recall, mean Average Precision with a 0.5 IOU threshold (mAP50), and mAP with a 0.5:0.95 IOU threshold (mAP50:95) smoothed curves of the best model after training. Training terminated at the 24th epoch. Note the differences between the *y*‐axis scales between the four graphs. As training proceeded, precision increased until leveling off while recall increased temporarily before decreasing. The two mean Average Precision values stayed relatively constant during training.

During training, the loss calculated for the three main tasks (box placement, object detection, and object classification) all decreased over the 24 training epochs, while during validation only the loss for object detection increased (Figure 4). This finding, along with a decrease in Recall, mAP50, and mAP50:95 over the 24 epochs, suggested that overfitting occurred during the training period. This diagnostic was considered acceptable as these metrics only decreased by a range of 0.005 to 0.02 (Figure 5). After the validation portion of the training script was completed, the model achieved a Precision value of 0.908 and Recall value of 0.942 across both classes. In other words, 90.8% of the positives detected were correctly identified and 94.2% of all possible ground truth positives were correctly detected. A mAP50 value of 0.921 was achieved while the mAP50:95 value was 0.523. The model's performance of each class was also evaluated using these metrics (Table 3). Separately, the model was evaluated with these same metrics on a test set that was not “seen” by the algorithm during the training or validation steps. On the test set, the model achieved an overall Precision value of 0.933 and Recall value of 0.931. The test set mAP50 value was 0.908 and its mAP50:95 value was 0.488. The metrics for each specific class were also evaluated (Table 4).

**TABLE 3:** Validation metrics after training and validating the model.

Class	Precision	Recall	mAP50	mAP50:95
All	0.908	0.942	0.921	0.523
Philodina	0.995	1.000	0.995	0.665
Cryptomonas	0.821	0.884	0.847	0.381

*Note:* The rows represent the performance of the overall model, the model's performance on just the *Philodina* class, and just the *Cryptomonas* class.

**TABLE 4:** Validation metrics after testing the model on unseen, labeled data.

Class	Precision	Recall	mAP50	mAP50:95
All	0.933	0.931	0.908	0.488
Philodina	1.000	1.000	0.995	0.669
Cryptomonas	0.867	0.862	0.820	0.306

*Note:* The rows represent the performance of the overall model, the model's performance handling the *Philodina* class, and its performance handling the *Cryptomonas* class.

## 4 Discussion

The PrED model performs well in its detection and classification tasks as can be seen with the high Precision, Recall, and mAP50 values achieved in both validation and testing (Tables 3 and 4). Because of PrED's strong performance with these underlying tasks, its ability to detect encounters and estimate prey density and abundance performs equally well. Looking at the class specific metrics, the model struggles more with falsely labeling objects as *C. erosa* and missing potential ground truth positives compared to how the model handles the *Philodina* class (Tables 3 and 4). While these diagnostics for *C. erosa* detection are lower than those for both *Philodina* and the overall model, these values for *C. erosa* are still strong enough to be useful (> 80%, Tables 3 and 4).

The intended use case for PrED is to help community ecology researchers save time and resources by finding the points in their videos where potential encounters occurred. In its current state, this model should not be considered a fully automated encounter frequency estimation model, but instead should be thought of as a semi‐automated tool. The encounter log the model outputs includes the timestamps of potential encounters so users can skip to those moments in their videos rather than spend time scrubbing through every frame. For context, the inference script is able to process a video with 2341 frames on a MacBook Pro with a M4 Pro chip and 24 GB of RAM in about 5 min and 48 s (Figure 2c). In contrast, annotating a video of this length took three to four days to complete.

While it resulted in more false positives, sensitivity was prioritized over precision to maximize true positives detected by the model. One issue of note for users, however, is that the encounter list the model outputs may be inflated due to SORT's limitations in handling occlusions. As a *C. erosa* encounters the *Philodina* sp., it is often directed underneath or over the rotifer because of movement in the feeding field. Therefore, one “Encounter!” is recorded as the individual approaches the rotifer and another is recorded after the individual reappears as it moves away. Future versions will drill down on this issue and explore better tracking implementations, such as models that can track through occlusions (Wojke et al. 2017). These models, however, tend to require the objects to be visually distinct from each other, which is unfortunately not a luxury when using environmentally realistic densities of *C. erosa* individuals (Wojke et al. 2017). While these limitations should certainly be kept in mind, the utility of this model lies in its ability to truncate a video into timestamps of interest for later review by cutting out sections where no potential encounters occur. The PrED model, therefore, has the potential to be a useful time saver for ecologists reviewing large video datasets, while also providing the foundation for more advanced, automated species interaction video analysis models in the future.

Having a comprehensive understanding of how plankton interact in a given ecosystem requires studying complex assemblages and community dynamics across large spatial and temporal scales (Carroll et al. 2019; Pringle and Hutchinson 2020; Bandara et al. 2021; Suraci et al. 2022; Jiang et al. 2023; Yi et al. 2024). These complexities, however, require large datasets to be collected and analyzed, both of which can only be accomplished with even larger amounts of time, money, and manpower. These limitations have already started to be alleviated by integrating machine learning techniques into studies on plankton functional traits, community assemblage, and distribution (Orenstein et al. 2022). Building off these integrations by incorporating computer vision models would not only further alleviate the limitations of traditional methods but would also create the possibility of directly observing biodiversity and community dynamics. Current work on directly observing plankton biodiversity and assemblage using in situ devices that detect and classify plankton species demonstrates the potential of expanding these devices' capabilities to include detection and classification of interactions (Pollina et al. 2022). While it will not be an easy feat to create an in situ device capable of observing and cataloging both community assemblage and dynamics, relatively basic models like PrED lay the groundwork for achieving a realistic, comprehensive understanding of plankton communities.

By cultivating a deeper knowledge of how microscopic planktonic interactions collectively drive large scale aquatic ecosystem community dynamics, managers will have more scientific resources at their disposal when making critical decisions regarding the ecosystem services they are charged with overseeing (Marrasé et al. 1990; Kiørboe 2008). These services will undoubtedly be affected as ecosystems and the organisms within them adapt to new climatic and environmental conditions (Traill et al. 2010; Wollrab et al. 2021). In order for managers to make decisions regarding the preservation of these services, it is imperative that ecologists provide them with a comprehensive and realistic picture of how the species of an ecosystem contribute to its functioning. For example, using CV/ML tools to get a more realistic understanding of which species of zooplankton are interacting the most with harmful algal species in a freshwater lake will be useful to water quality managers when monitoring the health of their ecosystems (Jeppesen et al. 2011; Ladds and Gobler 2025). A greater appreciation of the effect of species interaction dynamics, with realistic estimates of consumers' functional responses, would allow for more robust forecasts of future states for algal blooms.

While achieving this ideal system requires many more hours of work and iteration, this study has laid the groundwork to bring it to fruition. In the near future, the PrED model and the dataset on which it was trained will save aquatic ecologists time and resources when analyzing planktonic predator–prey interactions. In the long term, the PrED model will be the start of aquatic ecologists fully understanding the nuances of plankton interactions and aid in normalizing the integration of CV/ML techniques and technologies into the study of species interactions and ecology as a whole. Overall, our aim is that this study demonstrates that, even with no CV/ML or computer science background, incorporating these cutting edge tools into ecological work is not only possible but also within reach. In adapting these tools to work with specific ecological applications, the questions that were too complex for traditional methods will be answerable with empirical studies.

## Author Contributions

**Braden Charles DeMattei:** conceptualization (lead), data curation (lead), formal analysis (lead), investigation (lead), methodology (lead), project administration (lead), visualization (lead), writing – original draft (lead), writing – review and editing (equal). **Stephanie E. Hampton:** conceptualization (supporting), project administration (supporting), resources (lead), supervision (lead), writing – original draft (supporting), writing – review and editing (equal).

## Disclosure

*Statement on inclusion*: While this study was the product of a small team, it was also the result of attending a workshop comprised of an international group of instructors and students where everyone's input and opinions were considered and appreciated. The authors support inclusion, open science, and accessibility for all.

## Conflicts of Interest

The authors declare no conflicts of interest.

## Data Availability

The annotated video frame dataset used is available on HuggingFace at https://huggingface.co/datasets/bcdemattei/plankton_interaction_videos. The python scripts used in running the PrED model are available at https://github.com/bcdemattei/PrED‐Model.
